# The role of glutathione peroxidase 4 in the progression, drug resistance, and targeted therapy of non-small cell lung cancer

**DOI:** 10.32604/or.2024.054201

**Published:** 2025-03-19

**Authors:** JIAHENG WEI, LIANGMING ZHU

**Affiliations:** 1 School of Clinical Medicine, Shandong Second Medical University, Weifang, 261000, China; 2 Department of Thoracic Surgery, Jinan Central Hospital, Shandong University, Jinan, 250000, China

**Keywords:** Non-small cell lung cancer (NSCLC), Glutathione peroxidase 4 (GPX4), Drug resistance, Inhibitor, Biological function

## Abstract

Lung cancer is one of the main causes of cancer-related deaths globally, with non-small cell lung cancer (NSCLC) being the most prevalent histological subtype of lung cancer. Glutathione peroxidase 4 (GPX4) is a crucial antioxidant enzyme that plays a role in regulating ferroptosis. It is also involved in a wide variety of biological processes, such as tumor cell growth invasion, migration, and resistance to drugs. This study comprehensively examined the role of GPX4 in NSCLC and investigated the clinical feasibility of targeting GPX4 for NSCLC treatment. We discovered that GPX4 influences the progression of NSCLC by modulating multiple signaling pathways, and that blocking GPX4 can trigger ferroptosis and increase the sensitivity to chemotherapy. As a result, GPX4 represents a prospective therapeutic target for NSCLC. Targeting GPX4 inhibits the development of NSCLC cells and decreases their resistance to treatment.

## Introduction

Based on the latest relevant literature, as of 2022, lung cancer remains the leading cause of cancer-related fatalities, with an increasing incidence annually. Lung cancer is responsible for the highest number of cancer-related fatalities, and its incidence is increasing annually [1–5]. The non-small cell lung cancer (NSCLC) histological subtype is the most prevalent, accounting for an estimated 85% of all lung malignancies. In contrast, small cell lung cancer (SCLC) accounts for approximately 15% of the total number of lung cancer cases [3,4,6]. The 5-year survival rate for NSCLC is a mere 23%, and the majority of patients are diagnosed at an advanced stage [7]. The advancement of medicine has brought about significant progress in treating NSCLC, including in the fields of surgery, radiotherapy, chemotherapy, immunotherapy, and targeted therapy [4,8–10]. However, the prognosis for patients with advanced lung cancer remains unsatisfactory. Therefore, elucidating the mechanisms of NSCLC occurrence and development and identifying new prognostic markers are paramount for clinical diagnosis, treatment, and improved patient prognosis.

The antioxidant enzyme glutathione peroxidase 4 (GPX4), with a molecular mass of around 20–22 kDa, demonstrates a high level of specificity in its ability to catalyze the conversion of lipid hydroperoxides into corresponding alcohols [11]. Structurally, GPX4 is a monomeric selenoprotein that incorporates selenocysteine at its catalytic site [11]. The genomic organization of the GPX4 gene comprises seven exons and six introns, which encode the mitochondrial (mGPX4), cytosolic (cGPX4), and nuclear (nGPX4) isoforms [12]. These isoforms exhibit specific cellular localizations and functions; mGPX4 and cGPX4 are predominantly involved in cellular protection across various cell types, whereas nGPX4 is implicated in the terminal stages of spermatogenesis [12].

The principal biological function of GPX4 is detoxifying lipid hydroperoxides, thereby preserving the integrity of the cell membrane against oxidative insult [13]. This detoxification is facilitated by using reduced glutathione (GSH) as an electron donor, which is converted to lipid alcohols (PL-OH) from the reactive lipid hydroperoxides (PL-OOH), during which GSH oxidizes to glutathione disulfide (GSSG) [14]. The participation of GPX4 in this antioxidant pathway is crucial in inhibiting ferroptosis, as a reduction in GPX4 activity can result in uncontrolled lipid peroxidation and the consequent initiation of ferroptosis.

GPX4 is essential in the cellular antioxidant defense system [13,15–18]. Other antioxidant enzymes with similar functions to GPX4 include superoxide dismutases (SOD), catalases (CAT), glutathione S-transferases (GST), glutathione reductases (GR), thioredoxin peroxidases (TPx). SOD scavenges harmful superoxide radicals, whereas CAT decomposes hydrogen peroxide into water and oxygen, thereby reducing its toxicity [19]. GST metabolizes glutathione, which aids in the antioxidant defense against peroxides [20]. GR helps to maintain lowered GSH levels within the cell, as GSH serves as a cofactor for GPX4 and other antioxidant enzymes [21]. TPx’s antioxidant activity can maintain cellular redox balance [22]. These antioxidant enzymes collaborate to shield the cell against oxidative harm while also preserving regular physiological functions.

The objective of this study is to investigate the role of GPX4 in NSCLC, with a particular focus on its molecular mechanisms in tumor advancement and resistance to drugs. As a key antioxidant enzyme, GPX4 not only regulates the ferroptosis process but also plays a key role in tumor growth, invasion, and migration. Studying the molecular mechanisms of GPX4 has important clinical value for developing new treatment strategies and improving treatment effects.

Emerging evidence indicates that targeting GPX4 to enhance ferroptosis has potential as a viable therapeutic approach, especially in the field of cancer treatment [23,24]. In light of this, this study presents an overview of GPX4’s biological significance in NSCLC, with an emphasis on its key roles in drug resistance and targeted therapy.

## Biological Functions of GPX4

The intracellular antioxidant enzyme GPX4 aids in the elimination of lipid peroxidation products and the mitigation of oxidative stress [25,26]. According to research, GPX4 plays a critical role in ferroptosis regulation [26]. Additionally, GPX4 maintains membrane integrity, participates in tumorigenesis, and induces drug resistance [27–30].

### Cell protection

GPX4 plays a crucial role in cellular protection. Its antioxidant activity and cell membrane maintenance are key mechanisms for safeguarding cells against oxidative damage [31,32]. In its role as an antioxidant enzyme, GPX4 mainly aids in the elimination of intracellular lipid peroxides, protecting cells from oxidative stress-induced damage and death, thereby maintaining cell membrane stability and integrity [33–35]. In addition, GPX4 is also essential for maintaining the normal physiological function of red blood cells. Since red blood cells are sensitive to oxygen, GPX4 can reduce oxidative stress by breaking down lipid peroxides to prevent damage to the red blood cell membrane during blood circulation [36–38].

### Tumor development

GPX4 is crucial in regulating oxidative stress, maintaining cell membrane stability, and inhibiting ferroptosis, making it a significant therapeutic target for anticancer treatment [24,39,40]. Tumor cells often exist in highly oxidative environments, where the antioxidative effects of GPX4 may contribute to their survival [41]. By suppressing ferroptosis, GPX4 counteracts tumor development and protects tumor cells [38]. GPX4 is crucial in regulating oxidative stress, maintaining cell membrane stability, and inhibiting ferroptosis, making it a significant therapeutic target for anticancer treatment [24,39,40]. Tumor cells usually exist in highly oxidative environments, where the antioxidative effects of GPX4 may contribute to their survival [41]. By suppressing ferroptosis, GPX4 protects tumor cells and contributes to tumor development [38,42]. Also, GPX4 expression levels are significantly correlated with anticancer treatment resistance, according to multiple studies [15,43–46].

## Molecular Mechanisms of GPX4 in NSCLC

**Figure 1:**
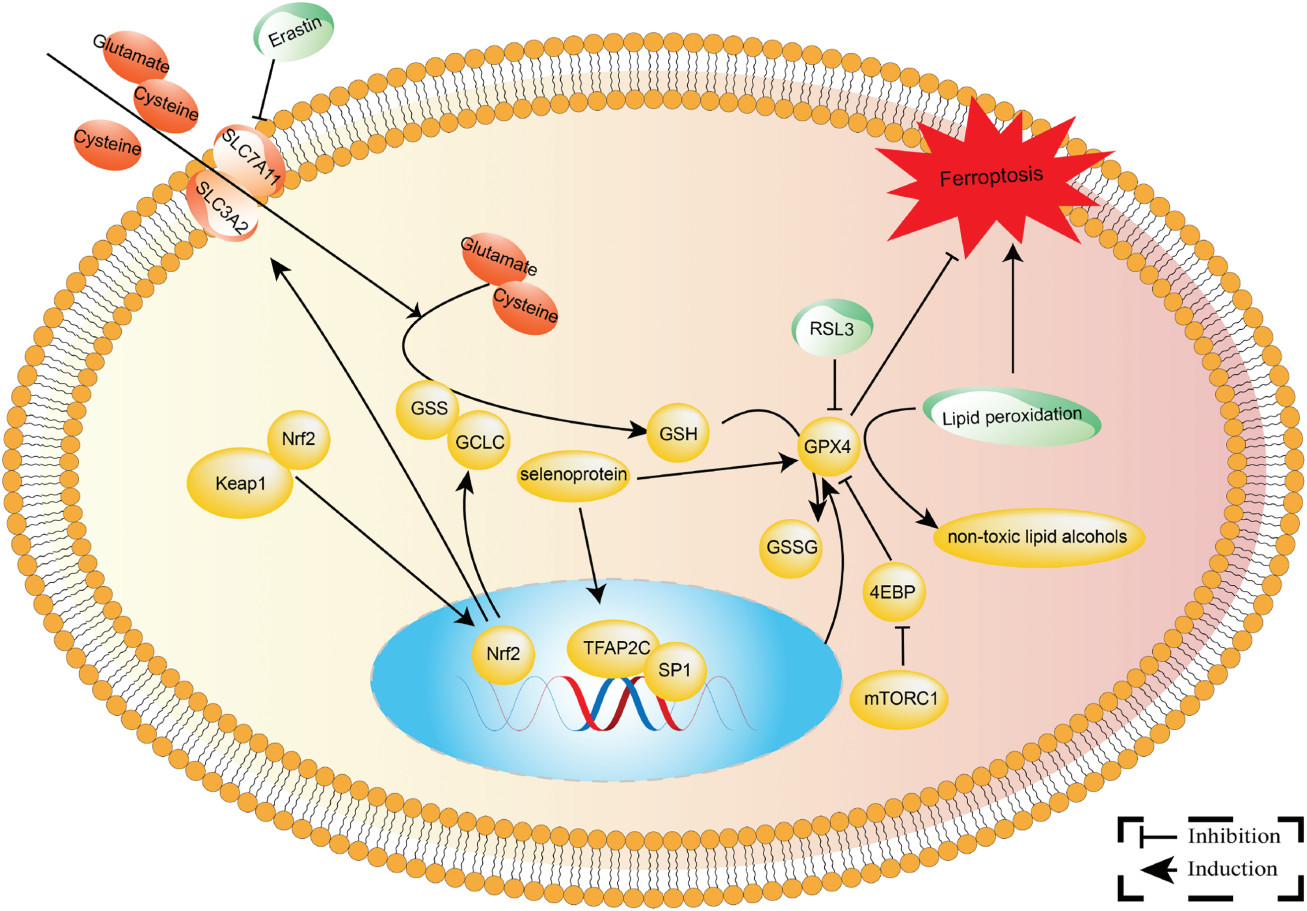
The molecular mechanisms involved in GPX4 in NSCLC. GPX4 is an essential antioxidant selenoprotein that converts dangerous lipid peroxides into innocuous lipid alcohols, thereby preserving cell membranes from oxidative damage. As an electron donor, Glutathione (GSH) is oxidized to glutathione disulfide (GSSG) and then reduced by glutathione reductase.

GPX4 eliminates lipid peroxides, safeguarding cell membranes against oxidative damage [47]. It plays pivotal roles in various physiological and pathological processes such as cell death, inflammatory responses, tumorigenesis, and drug resistance [48,49]. The expression of GPX4 is regulated by the AKT/STAT3 and Keap1/Nrf2 signaling pathways [50,51]. The AKT/STAT3 pathway enhances GPX4 stability by upregulating the expression of SLC7A11 [50]. Conversely, the Keap1/Nrf2 pathway downregulates GPX4 expression by inhibiting its transcription [51]. DNA methylation and histone acetylation may regulate GPX4 promoter transcription in cancer tissues. These regulatory mechanisms shed light on the involvement of GPX4 in cellular biology and NSCLC advancement (Fig. 1). Furthermore, activation of antioxidant pathways in NSCLC is regulated both before and after transcription, according to studies [51,52]. For example, activating the Keap1/Nrf2 pathway enhances the transcription of the GPX4 gene [51]. Notably, aberrant activation of antioxidant pathways is critical in the tumor-suppressive microenvironment, allowing tumor cells to elude immune surveillance and resist chemoradiotherapy [32–45].

## GPX4 and Lung Cancer Drug Resistance

**Figure 2:**
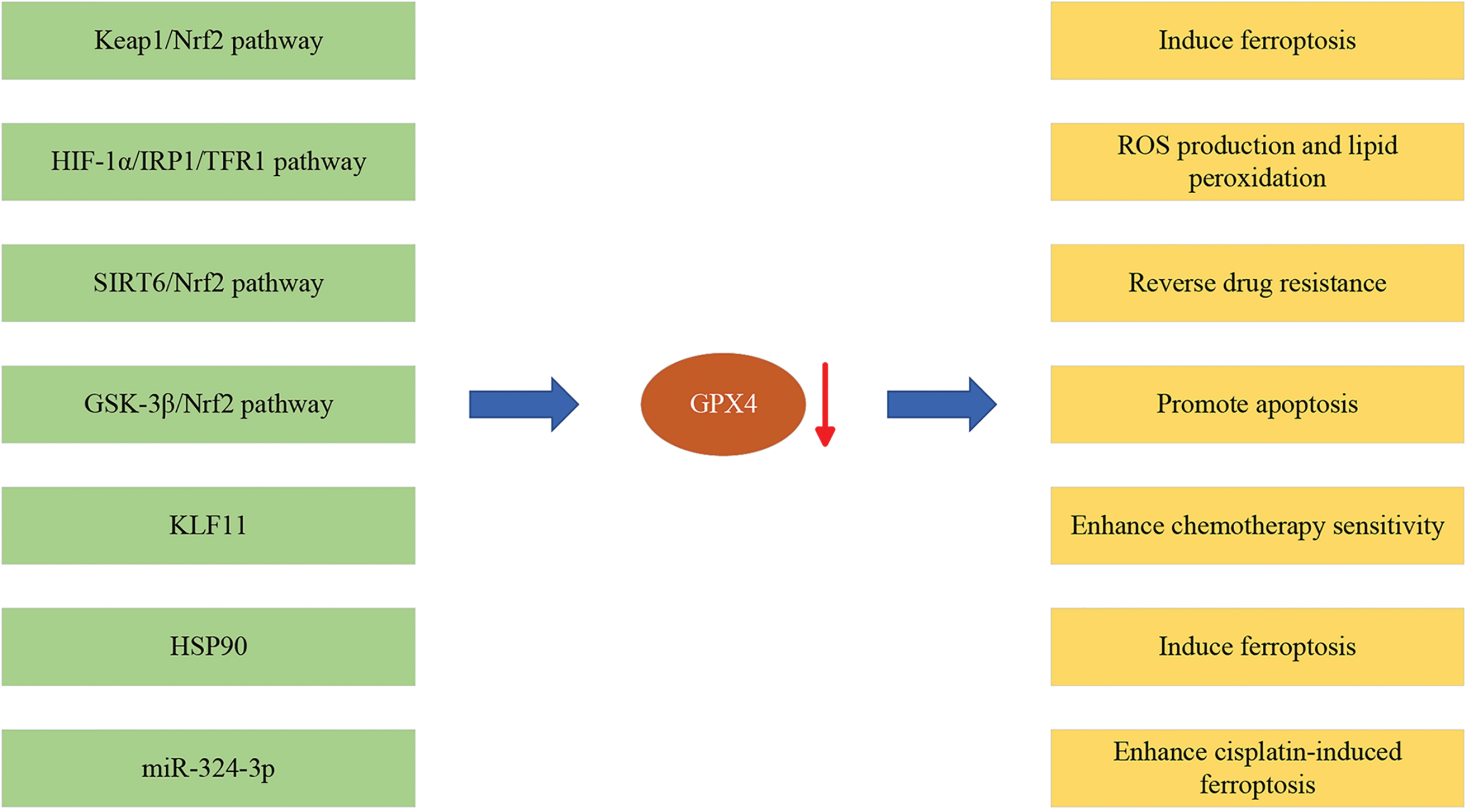
The roles of GPX4 in NSCLC development. Red arrow represents downregulation of GPX4 expression.

Chemoresistance is one of the primary reasons for disease progression in patients with late-stage lung cancer [53–55]. Effectively inhibiting the resistance of lung cancer cells can significantly prolong patient survival and potentially improve treatment outcomes. Recent research indicates that GPX4 is commonly overexpressed in NSCLC tissues and is closely associated with poor prognosis and chemotherapy sensitivity [30,56,57]. This finding suggests that suppressing GPX4 expression may be a potential strategy to overcome chemotherapy resistance in lung cancer [15,28,58]. A deeper understanding of the molecular mechanisms of lung cancer can help develop therapeutic strategies targeting GPX4 that can become an essential component of personalized treatment, offering novel prospects to enhance treatment efficacy and survival rates for patients with lung cancer (Fig. 2).

### GPX4 and chemoresistance

The expression of GPX4 exhibits a significant positive correlation with resistance to various chemotherapy drugs. Etoposide can induce ferroptosis in NSCLC cells; however, NSCLC cells inhibit ferroptosis by producing lactate through metabolic reprogramming, thus acquiring chemotherapy resistance [59]. Further research has indicated that lactate activates the p38-SGK1 signaling pathway, which thereby inhibits the ubiquitination and degradation of GPX4 by the E3 ubiquitin ligase NEDD4L to enhance GPX4 stability and activity, protecting NSCLC cells from lipid peroxidation damage.

Cisplatin promotes ferroptosis by inducing autophagy [15]. Mechanistically, autophagy can degrade the ferritin heavy chain (FTH1), thereby releasing more Fe^2+^. The above effects can be reversed by autophagy inhibitors such as 3-methyladenine (3-MA) or deferoxamine (DFO). GPX4 inhibitor RSL3 can enhance the sensitivity of lung cancer cells to cisplatin *in vivo* and *in vitro*.

GPX4 has been identified as a downstream target of miR-324-3p [28]. Upregulating miR-324-3p reduces GPX4 expression, making lung adenocarcinoma (LUAD) cells more sensitive to cisplatin. Additionally, the expression of GPX4 in LUAD is regulated by the transcription factor KLF11. KLF11 can inhibit GPX4 transcription by directly binding to the GPX4 promoter region, thereby promoting ferroptosis and enhancing chemotherapy sensitivity [60].

### GPX4 and targeted therapy resistance

Several studies suggest that targeted therapies for lung cancer, represented by epidermal growth factor receptor tyrosine kinase inhibitors (EGFR-TKIs), can effectively reduce tumor volume and significantly prolong patient survival [61–63]. Following treatment with EGFR-TKIs, the expression of GPX4 is significantly upregulated in LUAD cell lines that have developed resistance [15]. Additional research has demonstrated that EGFR-TKIs and the GPX4 inhibitor RSL3 can decrease the growth of EGFR-TKI-resistant LUAD cells when administered together.

In drug-resistant lung cancer cells, the expression level of Nrf2 is significantly elevated [58,64]. Silencing Nrf2 can reverse epithelial-to-mesenchymal transition (EMT) and decrease migration while increasing the sensitivity of cells to EGFR-TKIs [58]. Mechanistically, Nrf2 upregulates GPX4 and SOD2 expression to suppress reactive oxygen species (ROS) accumulation and cell death induced by EGFR-TKIs. Lapatinib (Lap) is a dual tyrosine kinase inhibitor that inhibits EGFR and ERBB2 [45]. In Lap-resistant NSCLC cells, the expression of GPX4 is upregulated. Silencing GPX4 can induce ferroptosis and increase NSCLC cells’ susceptibility to Lap. In a nude mouse xenograft model, inhibiting GPX4 inhibits tumor growth and enhances Lap’s anti-tumor activity [45].

## GPX4 Inhibitors

Studies reveal that GPX4 plays a significant role in the onset and progression of NSCLC [60,65]. Targeting GPX4 not only inhibits NSCLC cell growth but also effectively overcomes its drug resistance [66]. This finding offers new strategies and directions for NSCLC treatment, highlighting GPX4 as a potential therapeutic target. A deeper understanding of GPX4’s mechanisms in lung cancer can help develop more targeted and efficient treatment modalities in the future, leading to better clinical outcomes and survival rates for patients.

### Natural compounds targeting GPX4

Curcumin is a polyphenolic compound extracted from the turmeric plant that exhibits various biological properties such as anticancer, anti-inflammatory, and antioxidant effects [67–71]. Studies have demonstrated that curcumin can reduce the growth and stem cell-like characteristics of lung cancer cells by inducing ferroptosis through the downregulation of GPX4 expression [72,73].

Zerumbone, a natural compound derived from plants in the ginger family, has been discovered to effectively impede the advancement of NSCLC when used in conjunction with gefitinib (an EGFR tyrosine kinase inhibitor) [50]. Mechanistically, zerumbone and gefitinib can suppress lung cancer growth by inducing ferroptosis through the downregulation of GPX4, thereby enhancing the sensitivity of lung cancer cells to platinum-based chemotherapy drugs [50].

β-elemene (β-ELE), a natural compound obtained from *Curcuma wenyujin*, demonstrates significant anticancer activity in treating NSCLC [74]. Further research has revealed that β-ELE can significantly increase the expression of TFEB and promote lysosomal degradation of GPX4, leading to ferroptosis. Red ginseng polysaccharide (RGP), a bioactive compound found in the commonly used medicinal plant *Panax ginseng* C. A. Meyer (Araliaceae), triggers ferroptosis by reducing the expression of GPX4, resulting in anti-tumor activities [75].

Timosaponin AIII (Tim-AIII) is a steroidal saponin extracted from the traditional Chinese medicinal herb *Anemarrhena asphodeloides* Bunge, known for its significant anticancer activity [76–79]. Tim-AIII has been found to induce ferroptosis, which inhibits the development and lung metastasis of subcutaneous tumour grafts [80].

Bufotalin (BT) is a natural compound isolated from toads that belongs to the bufadienolide class of compounds [81,82]. BT accelerates the degradation of GPX4, elevates intracellular Fe^2+^ levels, induces ferroptosis and lipid peroxidation, and thereby inhibits the proliferation of NSCLC cells [83].

Researchers found that valtrate (Val) dramatically reduces tumor size and weight in xenograft tumor models by raising cleaved caspase-3 expression and lowering SLC7A11 and GPX4 expression [84]. Feng et al. observed that the combined treatment of isoorientin (IO) and cisplatin markedly reduces the survival rate of drug-resistant lung cancer cells [85]. This effect is accomplished by elevating intracellular Fe^2+^ levels, lipid peroxides, and ROS while concurrently reducing GSH levels, thus triggering ferroptosis in cells. Mechanistically, IO induces ferroptosis by modulating the SIRT6/Nrf2/GPX4 signaling pathway, thereby reversing drug resistance in lung cancer.

Photodynamic therapy (PDT) is a therapeutic modality that utilizes photosensitizers to release ROS under laser irradiation to destroy tumor cells [86,87]. However, PDT may also induce DNA damage and repair responses, upregulating GPX4 expression and degrading ROS, thus inducing resistance in tumor cells to the treatment [88–91]. Dihydroartemisinin (DHA) is a compound extracted from the plant *Artemisia annua*, possessing anti-malarial and anti-tumor properties [92,93]. Studies indicate that DHA can enhance the release of ROS generated by PDT by inhibiting GPX4 and inducing iron death, thereby improving its cytotoxic effect on lung cancer cells [94].

The Qingrehuoxue formula (QRHXF) significantly inhibits tumor growth, angiogenesis, and EMT while inducing apoptosis and ferroptosis in tumor cells [64]. Mechanistically, QRHXF can regulate the p53 and GSK-3β/Nrf2 signaling pathways, thereby downregulating GPX4 expression and upregulating apoptosis-related markers. Table 1 presents a comprehensive overview of natural compounds that specifically target GPX4.

**Table 1:** Natural compounds targeting GPX4

Drug	Activator/Inhibitor	Target	Mechanism	Biological function	Ref.
Curcumin	Inhibitor	GPX4	Inhibit GPX4 and FSP1 expression in A549 CD133+ cells.	Induce ferroptosis and inhibit cancer cell selfrenewal capability.	[72]
	Inhibitor	GPX4	Upregulate ACSL4 expression, downregulate SLC7A11 and GPX4 expression.	Induce autophagy and ferroptosis and inhibit NSCLC cell growth.	[73]
Isoorientin	Inhibitor	GPX4	Modulate the SIRT6/Nrf2/GPX4 signaling pathway.	Promote ferroptosis, reverses drug resistance, and inhibit cancer cell growth.	[85]
Dihydroartemisinin	Inhibitor	GPX4	Inhibit GPX4 upregulation and increase ROS.	Trigger ferroptosis, inhibit LLC survival and induce apoptosis.	[94]
Bufotalin	Inhibitor	GPX4	Downregulate GPX4 protein levels, promote its ubiquitination and degradation.	Induces ferroptosis and cytotoxicity, inhibit A549 cell growth.	[83]
Valtrate	Inhibitor	GPX4	Decrease SLC7A11 and GPX4 expression, increase intracellular Fe^2+^ levels.	Reduce the viability and proliferation capacity of A549 and H1299 cells, increase cell apoptosis, and ROS generation.	[84]
Timosaponin AIII	Inhibitor	GPX4	Promote ubiquitination and degradation of GPX4 by binding with HSP90.	Induce G2/M phase arrest and ferroptosis, inhibit NSCLC cell proliferation and migration.	[80]
β-elemene	Inhibitor	GPX4	Promote lysosomal degradation of GPX4.	Induce ferroptosis and inhibit NSCLC cell growth.	[74]
Zerumbone	Inhibitor	GPX4	Inhibit phosphorylation of Akt and STAT3, downregulate SLC7A11 and GPX4 expression.	Inhibit lung cancer cell proliferation and migration, promote cell apoptosis and ferroptosis, reduce tumor vascular density.	[50]
Red ginseng polysaccharide	Inhibitor	GPX4	Induce lactate dehydrogenase release, inhibit GPX4 expression.	Inhibit lung cancer cell proliferation.	[75]

### Organic compounds targeting GPX4

Propofol is a widely used intravenous medication for surgical procedures and serves as a neurosystemic anesthetic [95–97]. It exhibits anti-tumor effects in NSCLC by inhibiting cell proliferation and migration while promoting apoptosis. Further investigation revealed that propofol can reduce the half-maximal inhibitory concentration (IC_50_) value of NSCLC cells to cisplatin, thus reversing chemoresistance [95]. Mechanistically, propofol upregulates the expression of miR-744-5p/miR-615-3p, suppressing the transcription of GPX4 and inducing ferroptosis.

Ammonium ferric citrate (AFC) blocks the cell cycle at the G2/M phase and induces apoptosis, inhibiting the proliferation and migration of NSCLC cells [98]. AFC significantly increases intracellular Fe^2+^ levels and promotes oxidative stress damage, inhibiting autophagy and inducing ferroptosis. Additionally, AFC regulates the GSS/GGT axis, influencing GSH metabolism and ferroptosis. Table 2 presents a comprehensive overview of organic compounds that specifically target GPX4.

**Table 2:** Small molecule compounds targeting GPX4

Drug	Activator/Inhibitor	Target	Mechanism	Biological function	Ref.
ENBS-ML210	Inhibitor	GPX4	Promote lipid peroxidation, decrease GPX4 expression, reverse hypoxia-induced inhibition of ferroptosis.	Inhibit NSCLC cell growth.	[66]
Trabectedin	Inhibitor	GPX4	Activate the Keap1/Nrf2 pathway to inhibit GPX4 transcription.	Induce ferroptosis and inhibit NSCLC cell growth.	[51]

### Small molecule compounds targeting GPX4

RSL3 is a small molecule inhibitor of GPX4 that exhibits significant anti-tumor effects against NSCLC *in vitro* and *in vivo* [99]. RSL3 induces ferroptosis in NSCLC cells by inhibiting GPX4 and regulating the Nrf2/ HO-1 pathway [99]. Further research has revealed that RSL3 disrupts the stability of autophagic flux and lysosomal membranes and increases intracellular levels of Fe^2+^, lipid peroxides, and ROS while decreasing the GSH levels. Therefore, developing a tumor-specific nanoparticle vector for RSL3, which targets GPX4 in NSCLC, is feasible. These vectors can be engineered with surface modifications incorporating tumor-targeting ligands to facilitate specific binding to NSCLC cells. Concurrently, endowing the nanoparticles with pH-responsive properties to induce drug release within the TME helps minimize off-target effects on healthy cells. Furthermore, optimizing nanoparticle dimensions and morphology is essential to enhance their permeability and accumulation within the tumor mass. Employing these strategies helps augment the therapeutic index of RSL3, potentially leading to a more efficacious treatment regimen for NSCLC.

Hu et al. synthesized a compound called ENBS-ML210 by chemically bonding the photosensitizer ENBS with the GPX4 inhibitor ML210 [66]. When exposed to 660 nm irradiation, ENBS-ML210 produces a large amount of O^2−^, which enhances the process of lipid peroxidation and leads to the degradation of GPX4. *In vivo* experiments confirmed the efficient accumulation of ENBS-ML210 in tumor tissues, inhibiting tumor growth upon light exposure without observable toxic side effects.

The marine organism *Ecteinascidia turbinate* is the source of the anticancer drug Trabectedin [51,100,101]. Although Ttrabectedin exhibited no negative effects on normal cells, it was found to greatly inhibit NSCLC development independent of alterations in the tumor suppressor gene *p53* [51]. Trabectedin increases Fe^2+^ levels in NSCLC cells by regulating the HIF1α/IRP1/TFR1 axis, leading to the formation of ROS and lipid peroxidation. Reducing GSH levels in NSCLC cells, Trabectedin downregulates GPX4 expression by activating the Keap1/Nrf2 axis. Table 3 presents a comprehensive overview of small molecule compounds that specifically target GPX4.

**Table 3:** Organic compounds targeting GPX4

Drug	Activator/Inhibitor	Target	Mechanism	Biological function	Ref.
Ammonium ferric citrate	Inhibitor	GPX4	Increase intracellular Fe^2+^ and ROS levels, decrease levels of SOD, ATP, and GPX, inhibit expression of autophagy-related factors, reduce intracellular glutathione (GSH) levels.	Induce ferroptosis and inhibit NSCLC cell growth.	[98]
Propofol	Inhibitor	GPX4	Upregulate miR7445p/miR6153p, inhibit GPX4 transcription.	Induce ferroptosis and inhibit tumor growth and cisplatin resistance.	[95]

However, pharmacological inhibition of GPX4 includes targeted inhibition of enzyme activity and intervention at the transcriptional level [102–104]. To accurately regulate GPX4 expression, the former necessitates the development of small molecule inhibitors that bind to the protein’s active site. In addition, inhibitors of the bromodomain and extra-terminal (BET) protein family can inhibit GPX4 expression by blocking its transcriptional activity [104].

## Future Directions and Current Issues of GPX4 in NSCLC

GPX4 protects cells from oxidative stress damage by clearing excess ROS in cells [25]. However, as a core gene in the biological process of ferroptosis, GPX4 overexpression is strongly associated with the advancement of different types of malignancies, such as NSCLC [40,56,58,105–108]. Therefore, future studies can deeply analyze the expression level of GPX4 in different subtypes and stages of NSCLC and explore its specific molecular mechanism. In terms of clinical application, more specific GPX4 inhibitors or activators should be developed and screened, and combined with other anticancer drugs should be considered to optimize the treatment strategy.

## Conclusion

The biological significance of GPX4 in NSCLC is clarified in this study, and its overexpression is substantially correlated with a poor prognosis. Inhibiting GPX4 can induce ferroptosis and decrease drug resistance, which provides a novel therapeutic strategy for NSCLC patients. In addition, there were some compounds that showed promise as GPX4 inhibitors, further verification is required to confirm their clinical efficacy.

## Data Availability

Data sharing not applicable to this article as no datasets were generated or analyzed during the current study.
